# Prevalence of Antibiotic-Resistant Bacteria ESKAPE among Healthy People Estimated by Monitoring of Municipal Wastewater

**DOI:** 10.3390/antibiotics10050495

**Published:** 2021-04-26

**Authors:** Masateru Nishiyama, Susan Praise, Keiichi Tsurumaki, Hiroaki Baba, Hajime Kanamori, Toru Watanabe

**Affiliations:** 1 Department of Food, Life and Environmental Sciences, Faculty of Agriculture, Yamagata University, Tsuruoka 9978555, Japan; susigie1@yahoo.com (S.P.); k-tsurumaki@tds1.tr.yamagata-u.ac.jp (K.T.); 2 Department of Infectious Diseases, Internal Medicine, Tohoku University Graduate School of Medicine, Sendai 9808574, Japan; hbaba48@med.tohoku.ac.jp (H.B.); kanamori@med.tohoku.ac.jp (H.K.)

**Keywords:** ESKAPE bacteria, healthy people, municipal wastewater treatment plant, hospital wastewater

## Abstract

There is increasing attention toward factors that potentially contribute to antibiotic resistance (AR), as well as an interest in exploring the emergence and occurrence of antibiotic resistance bacteria (ARB). We monitored six ARBs that cause hospital outbreaks in wastewater influent to highlight the presence of these ARBs in the general population. We analyzed wastewater samples from a municipal wastewater treatment plant (MWWTP) and hospital wastewater (HW) for six species of ARB: Carbapenem-resistant Enterobacteria (CARBA), extended-spectrum β-lactamase producing Enterobacteria (ESBL), multidrug-resistant Acinetobacter (MDRA), multidrug-resistant *Pseudomonas aeruginosa* (MDRP), methicillin-resistant *Staphylococcus aureus* (MRSA), and vancomycin-resistant Enterococci (VRE). We registered a high percentage of ARBs in MWWTP samples (>66%) for all ARBs except for MDRP, indicating a high prevalence in the population. Percentages in HW samples were low (<78%), and no VRE was detected throughout the study. CARBA and ESBL were detected in all wastewater samples, whereas MDRA and MRSA had a high abundance. This result demonstrated the functionality of using raw wastewater at MWWTP to monitor the presence and extent of ARB in healthy populations. This kind of surveillance will contribute to strengthening the efforts toward reducing ARBs through the detection of ARBs to which the general population is exposed.

## 1. Introduction

Antibiotic resistance (AR) has become one of the major health threats worldwide [1,2,3]. As concerns grow, there is increasing attention and interest toward factors that potentially contribute to AR apart from the clinical ones [2], as well as an interest in exploring the emergence and occurrence of antibiotic resistance bacteria (ARB) and antibiotic resistance genes (ARGs) [3]. The enormous health and economic impacts presented by AR result from the overuse of antibiotics in humans and animals, poor hygiene and sanitation, and inefficient prevention and control of infections in healthcare settings [4]. Notably, the indiscriminate use of antibiotics is a key factor contributing to the alarming increase in ARB [1]. Moreover, findings in the past decade indicate the presence of AR in animals and foods [5], and the presence of the contaminant resistome in the environment portends further increase in AR in humans [6,7]. Antibiotics are widely used not only in human medicine but also across veterinary and agricultural practices for various purposes [8]. Hence, there is high ARB and ARG abundance in human and animal feces [2]. Human microbiota can be altered and enriched with ARB, therefore, humans can be considered as a source of ARB and ARG [3]. AR in humans is acquired through two major pathways: Hospital- and community-based [9]. ARBs are especially common in hospitals where they are associated with nosocomial infections [10,11]. Nosocomial pathogens, which are often referred to as “ESKAPE’’ pathogens, include both gram-positive and gram-negative species characterized by potential drug resistance mechanisms and are common causes of life-threatening hospital-acquired infections [11]. For example, Enterococci are major nosocomial pathogens due to their natural-intrinsic resistance to several antimicrobials (e.g., penicillin, ampicillin, and most cephalosporins) and their capacity to quickly acquire virulence and multidrug resistance [10,12].

AR is a worldwide burden and resistance to last-line antibiotics, such as carbapenems, fluoroquinolones, glycopeptides, and third-generation cephalosporins, is on the rise posing the greatest threat to human health [13,14]. Resistance to these drugs has been detected in hospitals worldwide [14], and is increasingly being detected in community-acquired (CA) infections. Examples include methicillin-resistant Staphylococcus aureus (MRSA), multidrug-resistant Streptococcus pneumoniae, vancomycin-resistant enterococci (VRE), and multidrug-resistant Escherichia coli and *Acinetobacter baumannii* [1,7,15]. Furthermore, carbapenem-resistant *Acinetobacter baumannii* and *Pseudomonas aeruginosa*, and carbapenem-resistant and third-generation cephalosporin-resistant Enterobacteriaceae are classified as critical-priority bacteria, whereas VRE, together with MRSA, has a high priority on the WHO priority list of ARB [16].

The prevalence and incidence of CA resistance, however, is difficult to assess without an acceptable criterion [9] and due to several risk factors associated with the communal acquisition of ARB. For example, in CA-MRSA, prior hospitalization in the past 12 months is a risk, and adults who acquire MRSA in the hospital may remain colonized for extended periods [9]. Moreover, prior colonization with these bacteria and exposure to third-generation cephalosporins and fluoroquinolones was a prerequisite for the community-onset of ESBL acquisition in Thailand [4]. ARB prevalence in healthy individuals varies between geographical areas, occupations, type of food consumed, environment, and antibiotic use [4,17]. Moreover, underlying diseases, antibiotic use, and invasive procedures in health care are the top three evidence-based risk factors for ARB in humans [18]. Although ARB is a global challenge, local actions, as well as geographical surveillance measures, are necessary to reduce its spread [2,19,20].

Only a few studies capture various community-level risk factors for ARB with little quantified evidence on the effect of the community [18]. Measuring the extent of AR in community- and healthcare-associated infections is crucial to define the issue and measure the outcomes of any interventions [18]. The occurrence of ARB is usually reported in hospitals in the context of nosocomial infections. Conversely, repeated quantification and focusing on similar established risks could, in turn, lead to more studies on the same established risk areas rather than entirely novel areas. Thus, to further highlight local risk factors, studies could incorporate qualitative techniques, such as surveys and interviews. Analyzing untreated wastewater from wastewater treatment plants (WWTPs) can be an alternative method of testing hundreds of human specimens received from the community and can be used as a detection system for the emergence of new or rare ARB [21]. Furthermore, WWTPs are hot spots for ARB and their spread [6,10,22]. Regardless of the small number of wastewater samples used, the results published by Paulshuns et al. [21] were surprisingly representative of the diversity in all individual urban wastewater sources, ensuring representability while validating the applicability of a small sample as a screening tool for the presence of ARB in the human population. In light of this, we believe that the majority of ARB in WWTPs are likely to have derived from the presence of such bacteria in the general population since those infected with ARB will not show any symptoms due to low virulence. We, therefore, hypothesize that ARB are circulated in a healthy population without causing any epidemics, which may be reflected in WWTPs. To examine this hypothesis, we monitored ARB in municipal WWTPs (MWWTPs) of a typical urban area with a population of 0.7 million people in Japan throughout one year. This study, therefore, aims to discuss the prevalence of ARB in healthy people versus clinical settings through monitoring municipal (WW) and hospital wastewater (HW).

## 2. Results

**Figure 1:**
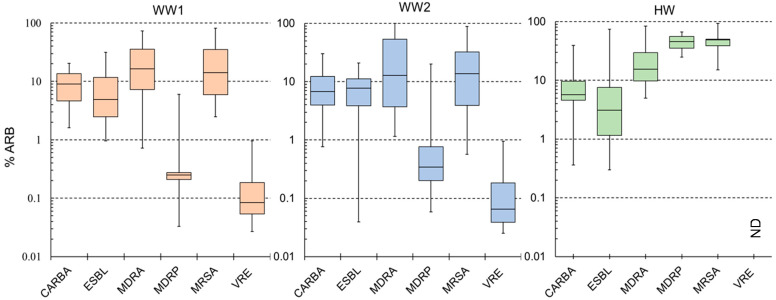
Summary of the results for ARB monitored in municipal (WW1 and WW2) and hospital wastewater. ND means no detection through the study period. CARBA, carbapenem-resistant *Enterobacteria*; ESBL, extended-spectrum β-lactamase producing *Enterobacteria*; MDRA, multidrug-resistant *Acinetobacter*, MDRP, multidrug-resistant *Pseudomonas aeruginosa*; MRSA, methicillin-resistant *Staphylococcus aureus*; VRE, vancomycin-resistant enterococci.

The percentage of ARB among the samples is summarized in Figure 1. The geometric mean of MRSA was the highest at all three sites, while MDRP had the lowest percentage at WW1 and WW2. The absolute mean values were higher at HW for MRSA, MDRP, and MDRA but there was no significant difference in geometric means. Although MDRA and MRSA had low percentages, their abundance was high in the positive samples compared to other ARB. At wastewater sites, MDRP had the least observed values both in detection frequency and the mean but showed the highest mean in HW. Based on the geometric mean and number of positive samples, the presence of CARBA, ESBL, and VRE was relatively stable at all three sites. In contrast, MDRA, MDRP, and MRSA percentages were highly variable, with more colonies detected in HW than in WW.

**Figure 2:**
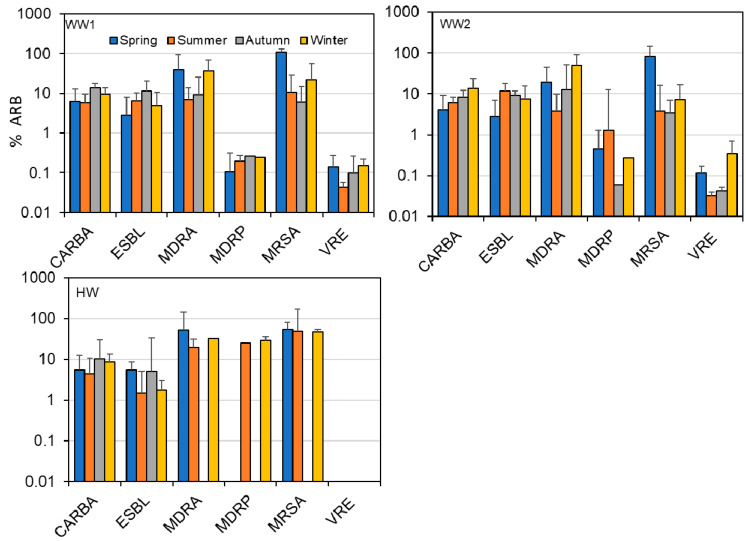
Percentage ARBs detected in each season from WW1, WW2, and HW during one year monitoring study in Sendai.

All the six studied ARBs were detected in WW samples. The most frequently detected ones were CARBA and ESBL at 100%, followed by MDRA and VRE at more than 79%. All but MDRP had a percentage above 66% at WW1 and WW2. In HW samples, only five ARBs were detected at a low frequency (less than 71%), and VRE was not detected (Table 1). Similar to those in WW samples, CARBA and ESBL percentages were high at > 71% in HW. MDRA and MDRP were less frequent, with 25.0 and 8.3%, respectively, in HW. The detection rate was significantly higher in WW sites than in HW sites (χ^2^ = 66.82, df = 10, *p* < 0.001) with pairwise significant difference between WW1 and HW, and WW2 and HW (*p* < 0.00001) but no significant differences between WW sites (*p* = 0.969). Although no significant differences were noted in the detection frequency between WW1 and WW2 (Appendix A Table A1), the detection frequency decreased by two samples for MRSA and the geometric means also decreased for CARBA and MDRA (~3 times), and MRSA (~11 times) (Table 1). Individual ARBs did not show significant variation between the sites and season, although there were notable differences in %ARB such as the absence of VRE in HW. MDRA, MDRP, and MRSA were detected in all seasons at WW sites, while these were not detected in HW samples during some seasons (Figure 2, Appendix A Table A1).

## 3. Discussion

We monitored ARB in WWTP and HW and found a higher prevalence of ARB in WWTP influent than that in HW. The selective agars used in this study are normally used for screening target ARB and further complicated tests are required for its exact identification. This study, aiming at simultaneous detection of six important ARB and comparison between WW and HW, allowed the rough isolation based on the screening media.

Wastewater is among the important reservoirs of AR, and the wastewater microbiome brings together bacteria from environmental, human, and animal origins [23]. All the ARB transported in wastewater ends up in WWTP, hence the high prevalence revealed in the present study. Although WWTPs receive influent from diverse sources, in the studied area, one sewer system (WW2) drained water from the general population, while the other (WW1) was the combined drainage for household wastewater and stormwater. Still, no significant differences were observed between the results for the two sites, implying that the effect of precipitation on ARB abundance and occurrence was negligible. What is clear in this study is that the origin of most ARB circulating in the studied environment was not from clinical sources but rather from the general population. ARB have been detected in healthy people, and food sources before, ESBL were detected in 75.5% of healthy food factory workers [4], and various prevalence rates were reported in healthy farm workers (77.3%), healthy animals (pigs 75.1%, chicken 38.8%, for Enterobacteriaceae), and fresh foods in Thailand [4]. As a niche for ARB and ARGs, the total microbial load in raw wastewater serves as an indicator of the ‘contamination index’ of the inflow and the required treatment effort [23]. The level and proportion of ARB in wastewater influent may reveal the presence and extent of ARB spread in healthy populations. The relative stability exhibited in the occurrence and abundance of CARBA, ESBL, and VRE in this study makes them a better target for this kind of monitoring.

The high percentage of MDRA, MDRP, and MRSA in HW indicated high occurrence in clinical settings. A similar scenario for a higher incidence of multidrug resistance (MDR) in hospital sewage than that in urban areas was observed in Coimbra, Portugal [24]. Most MDR bacteria, like MDRP, are opportunistic pathogens for humans and animals [25], and have been frequently reported in hospital outbreaks [26,27]. Consequently, hospitals are ecological niches for ARB and play a major role in the emergence and spread of these bacteria [11], especially for MDR species. The high abundance of MDRA, MDRP, and MRSA highlighted the level of colonization and spread of MDR bacteria in hospitals compared to that in the general population.

On the other hand, no VRE was detected in HW even though VRE accounts for the majority of human enterococcal infections and is a leading cause of hospital-acquired and multidrug-resistant bacterial infections [11,12]. This is consistent with no report of VRE isolation from hospitals in the prefecture, where this study was conducted, in the last five years, according to the Japan Nosocomial Infection Surveillance (https://janis.mhlw.go.jp/policy/index.html, accessed on 26 April 2021). This is also not surprising since a previous study on VRE revealed that VRE is more common in sewage samples, suggesting that the origin could be both healthy individuals and individuals in hospitals [28]. Hence, VRE may go unnoticed without causing any infections that require medical intervention. The variation in MDRA, MDRP, and MRSA bacterial population in this study, together with the size of the data, present a challenge for discussing the prevalence of MDR bacteria.

Some papers reported that microbial community and ARGs in wastewater found in sewer pipes were different from those in biofilm developed on the pipe wall [29,30]. Sewers are likely hotspots for AR accumulation and spread. However, the contribution of bacteria in biofilm to the percentage of ARB in wastewater samples should be limited unless a significant amount of biofilm is detached at once. The small variation of percentage of ARB throughout the year and a short traveling time (2–4 h) from discharge to sampling supports the limited contribution of detached biofilm. Some bacteria in the WW can grow during transportation in the sewer system. However, it seems unrealistic that such bacteria can acquire the antibiotic-resistance there, affecting the results obtained in this study, because (1) the horizontal gene transfer seldom occurs [31] even in an optimal condition like filter mating experiment (10^−7^ to 10^−3^) and (2) antibiotics in the municipal wastewater are generally low [32] and hardly cause selective pressure for vertical gene transfer. In this monitoring, we did not measure any parameter for water quality in WW and HW samples. We believe that ARB in wastewater are not significantly affected by water quality. Even if ARBs are affected, susceptible bacteria belonging to the same species should be affected equally, resulting in the constant percentage of ARB.

Although AR is a global challenge, local activity is necessary to reduce its spread [20,29]. In this study, wastewater from a hospital, as one of the hot spots of ARB, demonstrated a lower percentage of ARB than the municipal wastewater and no VRE was detected in HW. Moreover, the percentage of positive ARB samples was significantly higher in WW sites. This fact highlights the importance of ARB possessed by healthy people who never visit hospitals. These ARB ultimately find their way to the environment, contributing to the overall ARB burden. The evolution and spread of AR are complex processes resulting from the interplay of different and often confounding variables [19]. However, monitoring ARB in WWTP inflow, which can report a higher prevalence of important ARB and earlier occurrence of new ARB than the surveillance involving hospitals, may serve as a warning for communities and health workers, as well as aid in preventing the spread.

## 4. Materials and Methods

### 4.1. Sample Collection

This study was conducted for a year from February 2019 to February 2020 in Sendai, Miyagi prefecture Japan. The WWTP receives municipal wastewater from an approximate population of 735,700 at the flow rate of ca. 320,000 m^3^/day. The wastewater reaches the WWTP through the sewer system in 2 to 4 h. The WWTP received wastewater from two sewer systems (i.e., combined with and separated from urban drainage), hence two independent sampling points were set at WW1 and WW2 for combined and separated sewer systems, respectively. WW1 and WW2 samples were collected at the receiving well before the grift chamber. HW samples were taken from a sewer pipe connected to buildings for inpatients in a general hospital, which has 1200 beds and the number of newly hospitalized patients is ca. 900 to 1000 people/month. The average discharge from a hospital ward is 250 to 300 m^3^/day. The HW was not from special units for patients with outbreak diseases but from the wards for patients with general diseases and no treatment, including disinfection, was applied to it. WW1 and WW2 samples were taken as grab samples on the same day twice a month (*n* = 24 each). The HW sample was similarly taken in the same week but on a different day from the WW samples. All samples were collected in the afternoon when the average flow rate was observed at both sites. Water samples were collected in 50 mL sterile tubes and transported to the laboratory on ice. The samples were kept frozen at −80 °C until analysis.

The precipitation during the sampling days was less than 3 mm. Even if the precipitation dilutes WW1 samples from the combined sewer system, all bacteria, regardless of AR, should be equally diluted, resulting in no impact on the percentage of ARB. Although environmental factors in the pipe may affect the microbial community in WW, even in the short traveling time, bacteria in the same species should be equally impacted, with negligible change in the percentage of ARB.

### 4.2. Bacterial Enumeration

We focused on six species of ARB, including carbapenem-resistant *Enterobacteria* (CARBA), extended-spectrum β-lactamase producing *Enterobacteria* (ESBL), multidrug-resistant *Acinetobacter* (MDRA), multidrug-resistant *Pseudomonas aeruginosa* (MDRP), MRSA, and VRE, which are all known as ESKAPE, causing serious nosocomial infections all over the world (Cassini, 2019). Bacteria enumeration was performed on non-selective and selective agar for ARB and total bacteria, respectively. Preparation of the culture media for all the six tested ARB was performed using CHROMagar (CHROMagar, Paris, France), following the manufacturer’s protocols. CHROMagar VRE, CHROMagar MRSA, CHROMagar mSuper CARBA, CHROMagar ESBL, CHROMagar MDRA, and CHROMagar MDRP were used as selective media, while CHROMagar VRE without supplement, CHROMagar MRSA without supplement, CHROMagar orientation, CHROMagar *E. coli*, CHROMagar *Acinetobacter*, and CHROMagar *Pseudomonas* were used as non-selective media for VRE, MRSA, CARBA, ESBL, MDRA, and MDRP, respectively. Exactly 100 μL of the sample was streaked on selective and non-selective culture media. The plates containing the samples were incubated in aerobic conditions at 37 °C for 18–24 h. Produced colonies were counted and presented as the percentage of ARB (number of colonies on the selective media) in total bacteria (number of colonies on the respective non-selective media).

### 4.3. Statistical Analysis

Statistical analysis was conducted using Excel (Microsoft Office 2018) and R (version 4.0.2) on RStudio program (version 1.3.959). The Kruskal–Wallis test followed by Dunn’s with Bonferroni correction was used to compare differences in detection rate and %resistance among positive samples between the sites for each ARB. The data were grouped by season, i.e., spring (March–May), summer (June–August), autumn (September–November), and winter (December–February). Dunn’s test with Bonferroni correction was conducted on seasonal paired data of %resistance and detection rate for individual ARBs to evaluate the site-specific differences attributed to both seasonal and location factors. Whereas, Chi-square test for independence was used to evaluate the level of ARB presence among the three sites based on detection rate and geometric mean for all ARBs.

## Figures and Tables

**Table 1:** Detection rate of each ARB and its geometric mean of %resistance during the one-year study. CARBA, carbapenem-resistant *Enterobacteria*; ESBL, extended-spectrum β-lactamase producing *Enterobacteria*; MDRA, multidrug-resistant *Acinetobacter*; MDRP, multidrug-resistant *Pseudomonas aeruginosa*; MRSA, methicillin-resistant *Staphylococcus aureus*; VRE, vancomycin-resistant enterococci.

Site	ARB	Detection Rate (%)	Geometric Mean of % Resistance among Positive Samples
WW1	CARBA	100.0	8.0
	ESBL	100.0	5.1
	MDRA	95.8	13.9
	MDRP	33.3	0.3
	MRSA	75.0	14.3
	VRE	79.2	0.1
WW2	CARBA	100.0	7.0
	ESBL	100.0	4.9
	MDRA	91.7	13.5
	MDRP	33.3	0.5
	MRSA	66.7	11.1
	VRE	83.3	0.1
HW	CARBA	66.7	5.8
	ESBL	70.8	3.6
	MDRA	25.0	17.5
	MDRP	8.3	40.8
	MRSA	37.5	44.5
	VRE	0.0	Not available

## Appendix A

**Table A1:** Z scores and *p* values from Dunn test for pair comparisons with Bonferroni correction.

%ARB	Column1	Spring		Summer		Autumn		Winter	
	Comparison	*Z*	*P. unadj*	*Z*	*P. unadj*	*Z*	*P. unadj*	*Z*	*P. unadj*
MDRA	HW-WW1	0.217	0.828	1.006	0.314			−0.188	0.851
	HW-WW2	1.003	0.316	1.666	0.096			−0.391	0.696
	WW1-WW2	0.907	0.364	0.808	0.419	−0.384	0.701	−0.322	0.748
CARBA	HW-WW1	−0.144	0.885	0.264	0.791	−1.161	0.246	−0.325	0.745
	HW-WW2	0.314	0.753	0.017	0.987	0.013	0.989	−0.718	0.472
	WW1-WW2	0.512	0.608	−0.248	0.804	1.438	0.150	−0.425	0.671
ESBL	HW-WW1	0.766	0.444	−1.845	0.065	−1.498	0.134	−1.235	0.217
	HW-WW2	0.781	0.435	−2.992	0.003	−1.149	0.251	−1.918	0.055
	WW1-WW2	0.016	0.987	−1.203	0.229	0.390	0.696	−0.737	0.461
MDRP	HW-WW1			1.704	0.088			1.568	0.117
	HW-WW2			0.893	0.372			1.109	0.267
	WW1-WW2	−0.821	0.412	−1.148	0.251	0.795	0.427	−0.398	0.691
MRSA	HW-WW1	−1.203	0.229	1.192	0.233			0.729	0.466
	HW-WW2	−0.597	0.551	1.631	0.103			1.530	0.126
	WW1-WW2	0.635	0.525	0.551	0.581	0.298	0.766	0.980	0.327
VRE	HW-WW1								
	HW-WW2								
	WW1-WW2	0.154	0.877	0.591	0.555	1.891	0.059	−0.767	0.443
Detection rate	Comparison	*Z*	*P. unadj*	*Z*	*P. unadj*	*Z*	*P. unadj*	*Z*	*P. unadj*
MDRA	HW-WW1	−0.420	0.674	−1.261	0.207	−1.787	0.074	−1.576	0.115
	HW-WW2	−0.420	0.674	−1.261	0.207	−1.787	0.074	−1.576	0.115
	WW1-WW2	0.000	1.000	0.000	1.000	0.000	1.000	0.000	1.000
CARBA	HW-WW1	−1.545	0.122	0.000	1.000	−1.802	0.072	−1.287	0.198
	HW-WW2	−1.545	0.122	0.000	1.000	−1.802	0.072	−1.287	0.198
	WW1-WW2	0.000	1.000	0.000	1.000	0.000	1.000	0.000	1.000
ESBL	HW-WW1	−1.170	0.242	−1.170	0.242	−1.755	0.079	−1.521	0.128
	HW-WW2	−1.170	0.242	−1.170	0.242	−1.755	0.079	−1.521	0.128
	WW1-WW2	0.000	1.000	0.000	1.000	0.000	1.000	0.000	1.000
MDRP	HW-WW1	−1.312	0.190	−1.312	0.190	1.000	0.614	0.807	0.419
	HW-WW2	−1.817	0.069	−1.312	0.190	1.000	0.614	0.807	0.419
	WW1-WW2	−0.508	0.611	−0.508	0.611	1.000	1.000	0.000	1.000
MRSA	HW-WW1	−0.508	0.611	0.000	1.000	−0.712	0.477	−1.220	0.222
	HW-WW2	0.000	1.000	0.508	0.611	−0.203	0.839	−1.220	0.222
	WW1-WW2	0.635	0.525	0.551	0.581	0.508	0.611	0.000	1.000
VRE	HW-WW1	−1.310	0.190	−0.504	0.614	−1.813	0.070	−0.907	0.365
	HW-WW2	−1.310	0.190	−0.705	0.481	−1.310	0.190	−1.813	0.070
	WW1-WW2	0.000	1.000	−0.201	0.840	0.504	0.614	−0.907	0.365

Note: Blank column means the comparison for %ARB was not made due to no detection of the ARB in HW.

## References

[1] Gottlieb T., Nimmo G.R. (2011). Antibiotic resistance is an emerging threat to public health: An urgent call to action at the Antimicrobial Resistance Summit 2011. Med. J. Aust..

[2] Pruden A., Larsson D.G.J., Amézquita A., Collignon P., Brandt K.K., Graham D.W., Lazorchak J.M., Suzuki S., Silley P., Snape J.R. (2003). Management Options for Reducing the Release of Antibiotics and Antibiotic Resistance Genes to the Environment. Environ. Health Perspect..

[3] Rodriguez-Mozaz S., Chamorro S., Marti E., Huerta B., Gros M., Sànchez-Melsió A., Borrego C.M., Barceló D., Balcázar J.L. (2015). Occurrence of antibiotics and antibiotic resistance genes in hospital and urban wastewaters and their impact on the receiving river. Water Res..

[4] Boonyasiri A., Tangkoskul T., Seenama C., Saiyarin J., Tiengrim S., Thamlikitkul V. (2014). Prevalence of antibiotic resistant bacteria in healthy adults, foods, food animals, and the environment in selected areas in Thailand. Pathog. Glob. Health.

[5] Founou L.L., Founou R.C., Essack S.Y. (2016). Antibiotic Resistance in the Food Chain: A Developing Country-Perspective. Front. Microbiol..

[6] Manaia C.M. (2017). Assessing the Risk of Antibiotic Resistance Transmission from the Environment to Humans: Non-Direct Proportionality between Abundance and Risk. Trends Microbiol..

[7] Tacconelli E., Sifakis F., Harbarth S., Schrijver R., van Mourik M., Voss A., Sharland M., Rajendran N.B., Rodríguez-Baño J., Bielicki J. (2018). Surveillance for control of antimicrobial resistance. Lancet Infect. Dis..

[8] Holmes A.H., Moore L.S.P., Sundsfjord A., Steinbakk M., Regmi S., Karkey A., Guerin P.J., Piddock L.J.V. (2016). Understanding the mechanisms and drivers of antimicrobial resistance. Lancet.

[9] Charlebois E.D., Bangsberg D.R., Chambers H.F., Perdreau-Remington F. (2002). Population-Based Community Prevalence of Methicillin-Resistant Staphylococcus aureus in the Urban Poor of San Francisco. Clin. Infect. Dis..

[10] Hocquet D., Muller A., Bertrand X. (2016). What happens in hospitals does not stay in hospitals: Antibiotic-resistant bacteria in hospital wastewater systems. J. Hosp. Infect..

[11] Santajit S., Indrawattana N. (2016). Mechanisms of Antimicrobial Resistance in ESKAPE Pathogens. BioMed Res. Int..

[12] Ahmed M.O., Baptiste K.E. (2018). Vancomycin-Resistant Enterococci: A Review of Antimicrobial Resistance Mechanisms and Perspectives of Human and Animal Health. Microb. Drug Resist..

[13] Collignon P.J. (2002). 11: Antibiotic resistance. Infect. Dis..

[14] Mckenna M. (2013). The Last Resort: Health Officials Are Watching in Horror as Bacteria Become Resistant to Powerful Carbapenem Antibiotics—One of the Last Drugs on the Shelf. Nature.

[15] Cassini A., Högberg L.D., Plachouras D., Quattrocchi A., Hoxha A., Simonsen G.S., Colomb-Cotinat M., Kretzschmar M.E., Devleesschauwer B., Cecchini M. (2019). Attributable deaths and disability-adjusted life-years caused by infections with antibiotic-resistant bacteria in the EU and the European Economic Area in 2015: A population-level modelling analysis. Lancet Infect. Dis..

[16] Tacconelli E., Carrara E., Savoldi A., Harbarth S., Mendelson M., Monnet D.L., Pulcini C., Kahlmeter G., Kluytmans J., Carmeli Y. (2018). Discovery, research, and development of new antibiotics: The WHO priority list of antibiotic-resistant bacteria and tuberculosis. Lancet Infect. Dis..

[17] Van Duijkeren E., Hengeveld P., Zomer T.P., Landman F., Bosch T., Haenen A., van de Giessen A. (2016). Transmission of MRSA between humans and animals on duck and turkey farms. J. Antimicrob. Chemother..

[18] Chatterjee A., Modarai M., Naylor N.R., Boyd S.E., Atun R., Barlow J., Holmes A.H., Johnson A., Robotham J.V. (2018). Quantifying drivers of antibiotic resistance in humans: A systematic review. Lancet Infect. Dis..

[19] Pärnänen K.M.M., Narciso-da-Rocha C., Kneis D., Berendonk T.U., Cacace D., Do T.T., Elpers C., Fatta-Kassinos D., Henriques I., Jaeger T. (2019). Antibiotic resistance in European wastewater treatment plants mirrors the pattern of clinical antibiotic resistance prevalence. Sci. Adv..

[20] Pazda M., Kumirska J., Stepnowski P., Mulkiewicz E. (2019). Antibiotic resistance genes identified in wastewater treatment plant systems–A review. Sci. Total Environ..

[21] Paulshus E., Kühn I., Möllby R., Colque P., O’Sullivan K., Midtvedt T., Lingaas E., Holmstad R., Sørum H. (2019). Diversity and antibiotic resistance among *Escherichia coli* populations in hospital and community wastewater compared to wastewater at the receiving urban treatment plant. Water Res..

[22] Berendonk T.U., Manaia C.M., Merlin C., Fatta-Kassinos D., Cytryn E., Walsh F., Bürgmann H., Sørum H., Norström M., Pons M.-N. (2015). Tackling antibiotic resistance: The environmental framework. Nat. Rev. Microbiol..

[23] Manaia C.M., Rocha J., Scaccia N., Marano R., Radu E., Biancullo F., Cerqueira F., Fortunato G., Iakovides I.C., Zammit I. (2018). Antibiotic resistance in wastewater treatment plants: Tackling the black box. Environ. Int..

[24] Amador P.P., Fernandes R.M., Prudêncio M.C., Barreto M.P., Duarte I.M. (2015). Antibiotic resistance in wastewater: Occurrence and fate of *Enterobacteriaceae* producers of Class A and Class C β-lactamases. J. Environ. Sci. Health Part A.

[25] Sala A., Di Ianni F., Pelizzone I., Bertocchi M., Santospirito D., Rogato F., Flisi S., Spadini C., Iemmi T., Moggia E. (2019). The prevalence of *Pseudomonas aeruginosa* and multidrug resistant *Pseudomonas aeruginosa* in healthy captive ophidian. PeerJ.

[26] Breathnach A.S., Cubbon M.D., Karunaharan R.N., Pope C.F., Planche T.D. (2012). Multidrug-resistant Pseudomonas aeruginosa outbreaks in two hospitals: Association with contaminated hospital waste-water systems. J. Hosp. Infect..

[27] Magalhães M.J.T.L., Pontes G., Serra P.T., Balieiro A., Castro D., Pieri F.A., Crainey J.L., Nogueira P.A., Orlandi P.P. (2016). Multidrug resistant Pseudomonas aeruginosa survey in a stream receiving effluents from ineffective wastewater hospital plants. BMC Microbiol..

[28] Iversen A., Kuhn I., Franklin A., Mollby R. (2002). High Prevalence of Vancomycin-Resistant Enterococci in Swedish Sewage. Appl. Environ. Microbiol..

[29] Auguet O., Pijuan M., Borrego C.M., Rodriguez-Mozaz S., Triadó-Margarit X., Giustina S.V.D., Gutierrez O. (2017). Sewers as potential reservoirs of antibiotic resistance. Sci. Total Environ..

[30] Cayford B.I., Jiang G., Keller J., Tyson G., Bond P.L. (2017). Comparison of microbial communities across sections a corroding sewer pipe and the effects of wastewater flooding. Biofouling.

[31] Neela F.A., Nonaka L., Rahman M.H., Suzuki S. (2009). Transfer of the chromosomally encoded tetracycline resistance gene *tet(M)* from marine bacteria to *Escherichia coli* and *Enterococcus faecalis*. World J. Microb. Biot..

[32] Bengtsson-Palme J., Hammarén R., Pal C., Östman M., Björlenius B., Flach C.F., Larsson D.G.J. (2016). Elucidating selection processes for antibiotic resistance in sewage treatment plants using metagenomics. Sci. Total Environ..

